# Supplementary material for: Gamma-Glutamyl Transferase Plus Carcinoembryonic Antigen Ratio Index: A Promising Biomarker Associated with Treatment Response to Neoadjuvant Chemotherapy for Patients with Colorectal Cancer Liver Metastases
Source: Curr Oncol. 2025 Feb 18;32(2):117. doi: 10.3390/curroncol32020117 (PMC11854261; doi:10.3390/curroncol32020117)
Supplement: Supplementary file 1 [file curroncol-32-00117-s001.zip › curroncol-3425035-supplementary.pdf]

Supplementary Materials

1. Supplementary Figures

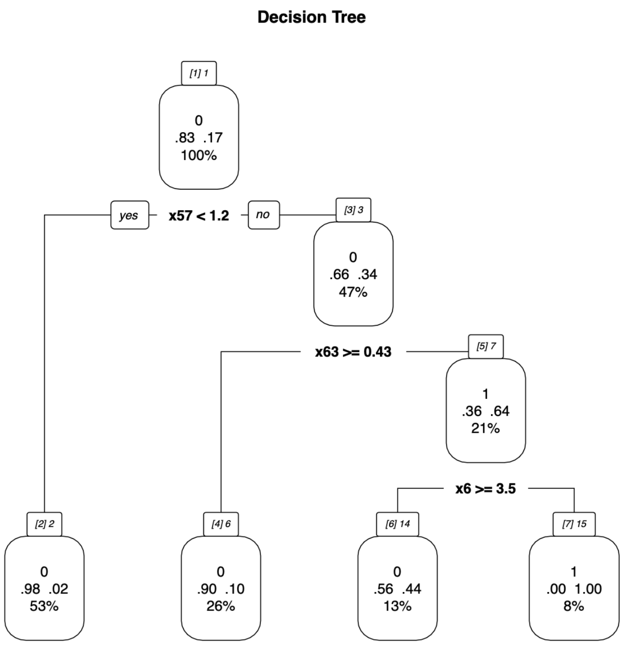

Figure S1. The variable selection process and decision boundaries of the decision tree model.

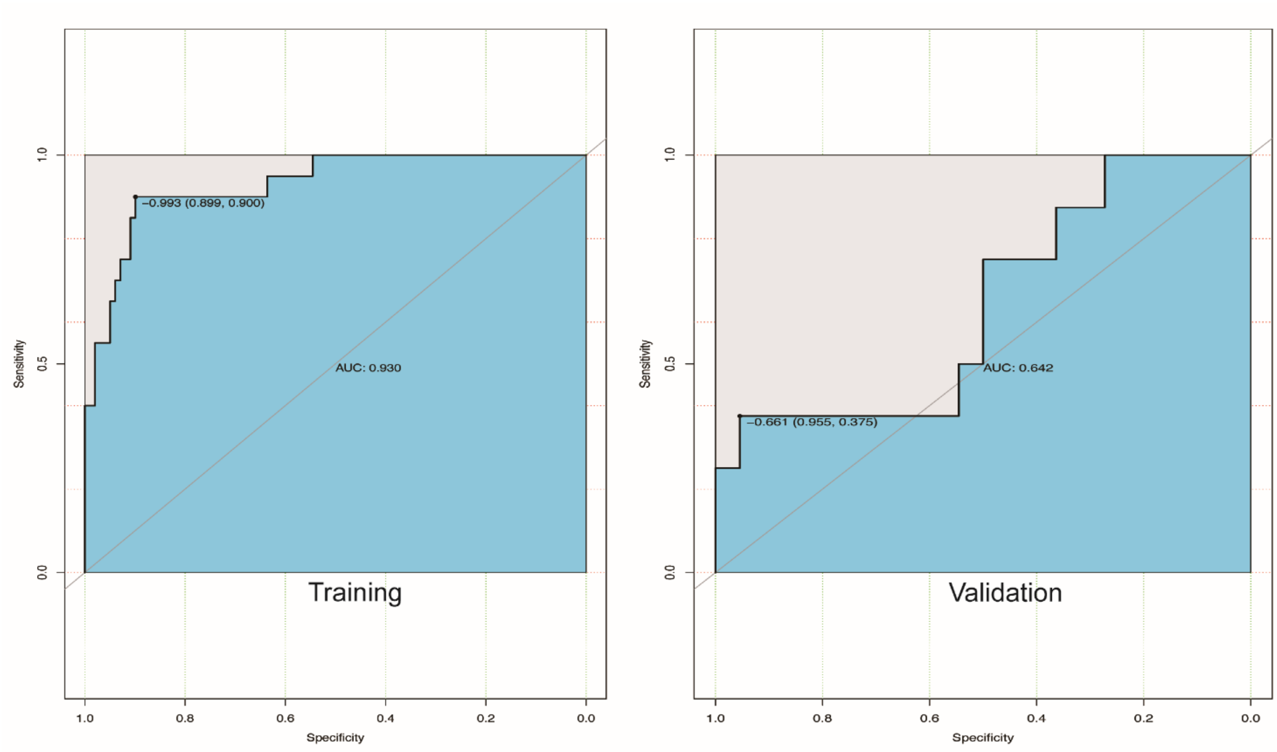

Figure S2. Receiver operating characteristic curves of support vector machine model for the training set (left) and validation set (right). AUC, area under the curve.

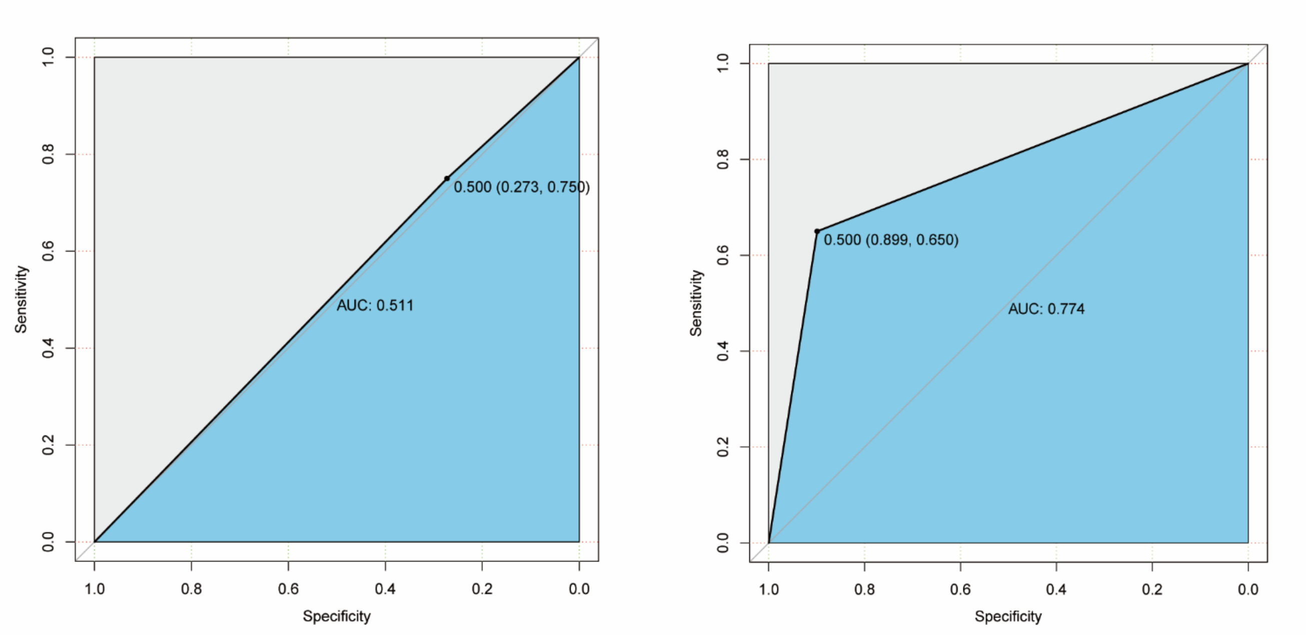

Figure S3. Receiver operating characteristic curves of AdaBoost model for the training set (left) and validation set (right). AUC, area under the curve.

2. Supplementary Tables

Table S1. Modified tumor regression grading scoring table

Classification standard	TRG 0	TRG 1	TRG 2	TRG 3	TRG 4	TRG 5
**Mandard**	-	No residual cancer cells	Minimal residual cancer cells	Fibrosis exceeding cancer cells	Cancer cells exceeding fibrosis	Almost no response
**Mandard's modified TRG tool**	-	5	4	3	2	1
**Dowrak/Rodel**	No response	<25% tumor area fibrosis	25%-50% tumor area fibrosis	>50% tumor area fibrosis	Complete remission	-
**Dowrak/Rödel's modified TRG tool**	1	2	3	4	5	-

TRG, Tumor regression grading.

Table S2. Comparison of clinical data among different groups

Variables	Training group (n=119)	Validation group (n=30)	*P*
**Demographic characteristics**			
Age			0.824
<=55	44 (36.9%)	10 (33.3%)	
>55	75 (63.1%)	20 (66.7%)	
Gender			0.904
Female	39 (32.8%)	9 (23.0%)	
Male	80 (67.2%)	21 (70.0%)	
**Clinicopathological features**			
BMI	23.9 (22.1, 26.2)	23.9 (20.6, 25.7)	1.000
No	65 (54.6%)	16 (53.3%)	
Yes	54 (45.4%)	14 (46.7%)	
ASA Classification			0.760
I	8 (6.7%)	1 (3.3%)	
II	96 (80.7%)	24 (80.0%)	
III	15 (12.6%)	5 (16.7%)	
Number of liver metastases	2.0 (1.0, 4.0)	3.0 (1.5, 5.0)	0.605[c]
CRS Score			0.842
1	14 (11.8%)	5 (16.7%)	
2	32 (26.9%)	9 (30.0%)	
3	64 (53.8%)	13 (43.3%)	
4	9 (7.6%)	3 (10.0%)	
Primary site			0.242
Colon	62 (52.1%)	12 (40.0%)	
Rectum	57 (47.9%)	18 (60.0%)	
Differentiation			0.758
Well	74 (62.2%)	20 (66.7%)	
Poor	45 (37.8%)	10 (33.3%)	

T Stage			0.213
T1-T2	108 (90.8%)	24 (80.0%)	
T3-T4	11 (9.2%)	6 (20.0%)	
N Stage			0.429
N0	32 (26.9%)	10 (33.3%)	
N1	87 (73.1%)	20 (66.7%)	
**Treatment information**			
Surgical sequence			1.000
Colorectal resection first	91 (76.5%)	23 (76.7%)	
Liver resection first	28 (23.5%)	7 (23.3%)	
Surgical procedures			0.355
Laparoscopy and laparoscopic assistance	18 (15.1%)	8 (26.7%)	
Laparoscopy	75 (63.0%)	15 (50.0%)	
Laparotomy	26 (21.8%)	7 (23.3%)	
Blood loss (mL)	200.0 (200.0, 500.0)	200.0 (125.0, 300.0)	0.079[c]
Operation time (min)	360.0 (300.8, 448.5)	390.0 (292.5, 447.5)	0.988
Intraoperative transfusion			0.980
No	95 (79.8%)	23 (76.7%)	
Yes	24 (20.2%)	7 (23.3%)	
Postoperative complication			0.550
No	60 (50.4%)	13 (43.3%)	
Yes	59 (49.6%)	17 (56.7%)	
Postoperative adjuvant chemotherapy			0.084
No	50 (42.0%)	7 (23.3%)	
Yes	69 (58.0%)	23 (76.7%)	
Postoperative hospital stays (Day)	10.0 (9.0, 14.0)	11.0 (9.0, 13.0)	0.633
**Laboratory tests**			
bPNI	52.3 (46.3, 56.0)	53.2 (47.4, 55.2)	0.833[c]
bNLR	2.4 (1.8, 3.3)	2.5 (1.9, 3.4)	0.809
bMLR	4.3 (3.1, 5.9)	4.2 (3.3, 5.4)	0.867

bPLR	140.6 (110.3, 192.9)	155.6 (129.8, 192.5)	0.468
bWBC	7.0 (5.7, 8.5)	6.5 (5.3, 8.1)	0.496
bANC	4.3 (3.5, 5.6)	4.2 (3.2, 5.4)	0.490
bAMC	0.4 (0.3, 0.6)	0.4 (0.3, 0.5)	0.471
bALC	1.8 (1.4, 2.3)	1.7 (1.4, 2.1)	0.796
bHb	140.0 (122.0, 149.0)	136.0 (122.5, 152.0)	0.634
bPLT	255.0 (211.2, 317.0)	277.0 (245.5, 314.0)	0.790
bPlasma D-dimer	0.3 (0.2, 0.7)	0.4 (0.3, 0.7)	0.810[c]
bALT	17.0 (11.0, 25.8)	17.0 (12.0, 25.0)	0.797[c]
bAST	22.5 (16.0, 48.0)	20.0 (17.5, 26.0)	0.777[c]
bLDH	175.0 (135.0, 224.2)	199.0 (161.5, 225.5)	0.551[c]
bGGT	30.0 (16.2, 53.5)	23.0 (17.5, 46.0)	0.312[c]
bALP	74.5 (55.0, 96.0)	77.0 (61.5, 88.0)	0.987[c]
bTBIL	8.4 (6.1, 11.0)	9.6 (6.4, 11.8)	0.526[c]
bDBIL	4.1 (3.0, 8.4)	4.0 (3.0, 5.7)	0.966[c]
bALB	44.0 (39.3, 45.7)	44.1 (40.9, 45.0)	0.857[c]
bCA19-9	27.6 (11.3, 102.0)	17.0 (10.5, 73.2)	0.447[c]
bCEA	12.3 (2.9, 47.1)	14.3 (4.0, 53.3)	0.533[c]
aPNI	51.8 (48.6, 54.9)	50.9 (47.3, 53.2)	0.143
aNLR	1.7 (1.3, 2.3)	1.7 (1.1, 2.6)	0.969
aLMR	3.7 (2.8, 4.8)	4.1 (3.0, 4.5)	0.913
aPLR	104.6 (82.4, 139.0)	111.5 (78.6, 134.0)	0.635
aWBC	5.4 (4.8, 6.5)	5.8 (4.1, 6.1)	0.310
aANC	3.0 (2.5, 3.8)	3.3 (2.4, 3.8)	0.807
aAMC	0.5 (0.4, 0.6)	0.4 (0.4, 0.5)	0.733
aALC	1.8 (1.5, 2.2)	1.7 (1.5, 2.1)	0.661
aHb	133.0 (124.2, 143.8)	130.0 (112.5, 137.5)	0.363
aPLT	190.0 (148.5, 231.8)	200.0 (155.5, 236.5)	0.676
aPlasma D-dimer	0.4 (0.3, 0.7)	0.5 (0.3, 0.7)	0.904
aALT	21.0 (14.0, 31.0)	26.0 (15.5, 42.5)	0.669

aAST	23.0 (18.0, 30.0)	28.0 (19.5, 37.5)	0.130[c]
aLDH	193.0 (166.5, 218.8)	199.0 (172.5, 223.2)	0.055
aGGT	35.5 (25.0, 62.0)	40.1 (29.0, 60.5)	0.859
aALP	79.5 (67.0, 98.5)	95.4 (77.3, 113.5)	0.090
aTBIL	9.0 (6.9, 12.5)	9.7 (7.3, 11.8)	0.351
aDBIL	3.7 (2.9, 4.5)	3.8 (2.9, 4.6)	0.782
aALB	42.7 (40.1, 45.3)	42.2 (39.5, 43.8)	0.147
aCA19-9	17.7 (9.5, 35.9)	14.2 (9.1, 30.8)	0.413[c]
aCEA	7.7 (3.4, 22.8)	5.9 (3.6, 19.2)	0.859[c]
rPNI	1.0 (0.9, 1.1)	1.0 (0.9, 1.0)	0.449[c]
rNLR	0.7 (0.5, 1.0)	0.7 (0.6, 0.9)	0.910
rLMR	0.9 (0.7, 1.1)	0.9 (0.7, 1.2)	0.780
rPLR	0.8 (0.6, 0.9)	0.7 (0.6, 0.8)	0.805
rWBC	0.8 (0.7, 1.0)	0.9 (0.7, 1.0)	0.667
rANC	0.7 (0.5, 0.9)	0.7 (0.6, 0.9)	0.925
rAMC	1.1 (0.9, 1.4)	1.2 (0.9, 1.4)	0.885
rALC	1.0 (0.9, 1.2)	1.0 (0.9, 1.3)	0.957
rHb	1.0 (0.9, 1.0)	0.9 (0.9, 1.0)	0.188
rPLT	0.7 (0.6, 0.9)	0.8 (0.6, 0.8)	0.656
rPlasma D-dimer	1.1 (0.6, 2.2)	1.3 (0.7, 2.0)	0.811[c]
rALT	1.1 (0.7, 2.2)	1.3 (0.7, 3.0)	0.440
rAST	1.0 (0.6, 1.5)	1.3 (0.7, 1.8)	0.511
rLDH	1.0 (0.8, 1.3)	1.0 (0.9, 1.2)	0.591[c]
rGGT	1.2 (0.9, 1.9)	1.3 (1.0, 2.6)	0.209[c]
rALP	1.0 (0.9, 1.5)	1.1 (0.9, 1.7)	0.306[c]
rTBIL	1.1 (0.8, 1.6)	1.1 (0.8, 1.9)	0.624
rDBIL	0.9 (0.5, 1.2)	0.9 (0.6, 1.1)	0.783[c]
rALB	1.0 (0.9, 1.1)	1.0 (0.9, 1.0)	0.152
rCA19-9	0.8 (0.3, 1.2)	1.0 (0.5, 1.2)	0.487[c]
rCEA	0.7 (0.2, 1.4)	1.6 (0.3, 6.6)	0.062[c]

a: after NAC; b: before NAC, [c]: Mann–Whitney U-test; r: ratio; BMI: body mass index; ASA: the American society of anesthesiologists; PNI: prognostic nutritional index; NLR: neutrophil-to-lymphocyte ratio; LMR: lymphocyte-to-monocyte ratio; PLR: platelet-to-lymphocyte ratio; WBC: white blood cell count; ANC: absolute neutrophil count; AMC: absolute monocyte count absolute lymphocyte count; Hb: hemoglobin; PLT: platelet; ALT: alanine aminotransferase; AST: aspartate aminotransferase; LDH: lactate dehydrogenase; GGT: gamma-glutamyl transferase; ALP: alkaline phosphatase; TBIL: total bilirubin; DBIL: direct bilirubin; ALB: albumin; CA19-9: carbohydrate antigen 19-9; CEA: carcinoembryonic antigen; CRS: comprehensive risk score.

Table S3. Coefficients and corresponding names of the 15 selected feature variables

Feature	Variable name	Coefficient	Feature	Variable name	Coefficient
PNI	x1	0.635474	rPLR	x46	0.007857
ALP	x16	-0.007680	rANC	x48	0.438790
aPNI	x22	0.000499	rGGT	x57	0.005605
aNLR	x23	0.148336	rALP	x58	0.015160
aLMR	x24	-0.00178	rCA19-9	x62	-1.887171
rCEA	x63	-0.007901	Primary site	x69	-0.260619
Age	x65	-0.011677	Preoperative comorbidities	x68	-0.073543
Sex	x66	-0.006668			

a: after NAC; r: ratio; PNI: prognostic nutritional index; NLR: neutrophil-to-lymphocyte ratio; LMR: lymphocyte-to-monocyte ratio; PLR: platelet-to-lymphocyte ratio; ANC: absolute neutrophil count; GGT: gamma-glutamyl transferase; ALP: alkaline phosphatase; CA19-9: carbohydrate antigen 19-9; CEA: carcinoembryonic antigen.

Table S4. Parameters of the feature variables identified by the logistic regression model and their corresponding names

Feature	Variable name	Training (n=119)	Validation (n=30)	OR (Univariable)	OR (Multivariable)
GGT	x15	50.0±52.0	22.9±20.9	0.97 (0.95-1.00, *P*=.020)	0.97 (0.95-0.99, *P* =.009)
aLMR	x24	3.9±1.5	4.7±2.0	1.32 (1.04-1.67, *P* =.021)	1.23 (0.95-1.60, *P* =.119)
aCEA	x42	37.4±100.1	6.8±9.2	0.94 (0.89-0.99, p=.028)	0.94 (0.89-1.00, *P* =.037)
Number of liver metastases	x67	3.7± 3.2	2.3±1.5	0.77 (0.61-0.98, *P* =.031)	0.81 (0.63-1.04, *P* =.100)

a: after NAC; OR: odds ratio; GGT: gamma-glutamyl transferase; LMR: lymphocyte-to-monocyte ratio; CEA: carcinoembryonic antigen.
